# Australian atmospheric pressure and sea level data during the 2022 Hunga-Tonga Hunga-Ha’apai volcano tsunami

**DOI:** 10.1038/s41597-024-02949-2

**Published:** 2024-01-23

**Authors:** Gareth Davies, Kaya Wilson, Ben Hague, Diana Greenslade, Daryl Metters, Paul Boswood, Sam Maddox, Sarah-Kate Dakin, Karen Palmer, Ben Galton-Fenzi, John French, Claire Kain

**Affiliations:** 1 Geoscience Australia, Symonston, Australia; 2 Bureau of Meteorology, Melbourne, Australia; 3 Queensland Department of Environment and Science, Brisbane, Australia; 4 Manly Hydraulics Laboratory, Sydney, Australia; 5 University of Tasmania, Hobart, Australia; 6 Australian Antarctic Division, Hobart, Australia; 7 Mineral Resources Tasmania, Rosny Park, Australia; 8 Present Address: AECOM, Canberra, Australia

**Keywords:** Physical oceanography, Natural hazards

## Abstract

On January 15, 2022, an ongoing eruption at the Hunga-Tonga Hunga-Ha’apai volcano generated a large explosion which resulted in a globally observed tsunami and atmospheric pressure wave. This paper presents time series observations of the event from Australia including 503 mean sea level pressure (MSLP) sensors and 103 tide gauges. Data is provided in its original format, which varies between data providers, and a post-processed format with consistent file structure and time zone. High-pass filtered variants of the data are also provided to facilitate study of the pressure wave and tsunami. For a minority of tide gauges the raw sea level data cannot be provided, due to licence restrictions, but high-pass filtered data is always provided. The data provides an important historical record of the volcanic pressure wave and tsunami in Australia. It will be useful for research on atmospheric and ocean waves associated with large volcanic eruptions.

## Background & Summary

On January 15, 2022, at approximately 04:15 UTC, an ongoing eruption at the Hunga-Tonga Hunga-Ha’apai volcano (184.615°E, 20.55°S) in Tonga produced a large explosion with global-scale effects not seen since the 1883 Krakatau volcanic eruption. These included global atmospheric pressure waves with a particularly prominent Lamb wave, seismic and acoustic waves, and a volcanic plume reaching an unprecedented 55 km height^1–4^. The ocean was perturbed by a combination of mass movements at the volcano, the explosion, and pressure gradients induced by the atmospheric waves, generating a tsunami that was observed globally. Tsunami runup heights reached 20 m in nearby Tonga, while peak-to-trough wave heights reached at least 3.4 m in the eastern Pacific and 1 m in the Atlantic Ocean^5–7^.

The Hunga-Tonga Hunga-Ha’apai volcano (henceforth HTHH volcano) is named after two small islands on the caldera’s northern rim^5^. Its explosive eruption is particularly significant as the first global-scale volcanic tsunami to be well recorded on modern atmospheric pressure and sea level sensor networks. Study of this event will enable better understanding of volcanic tsunami source processes, the dynamics of atmospheric Lamb waves, and the behaviour of volcanic tsunamis, with application to tsunami hazard assessment and risk mitigation, among other fields. For this reason, it is important to archive and facilitate access to observations of the event.

**Fig. 1:**
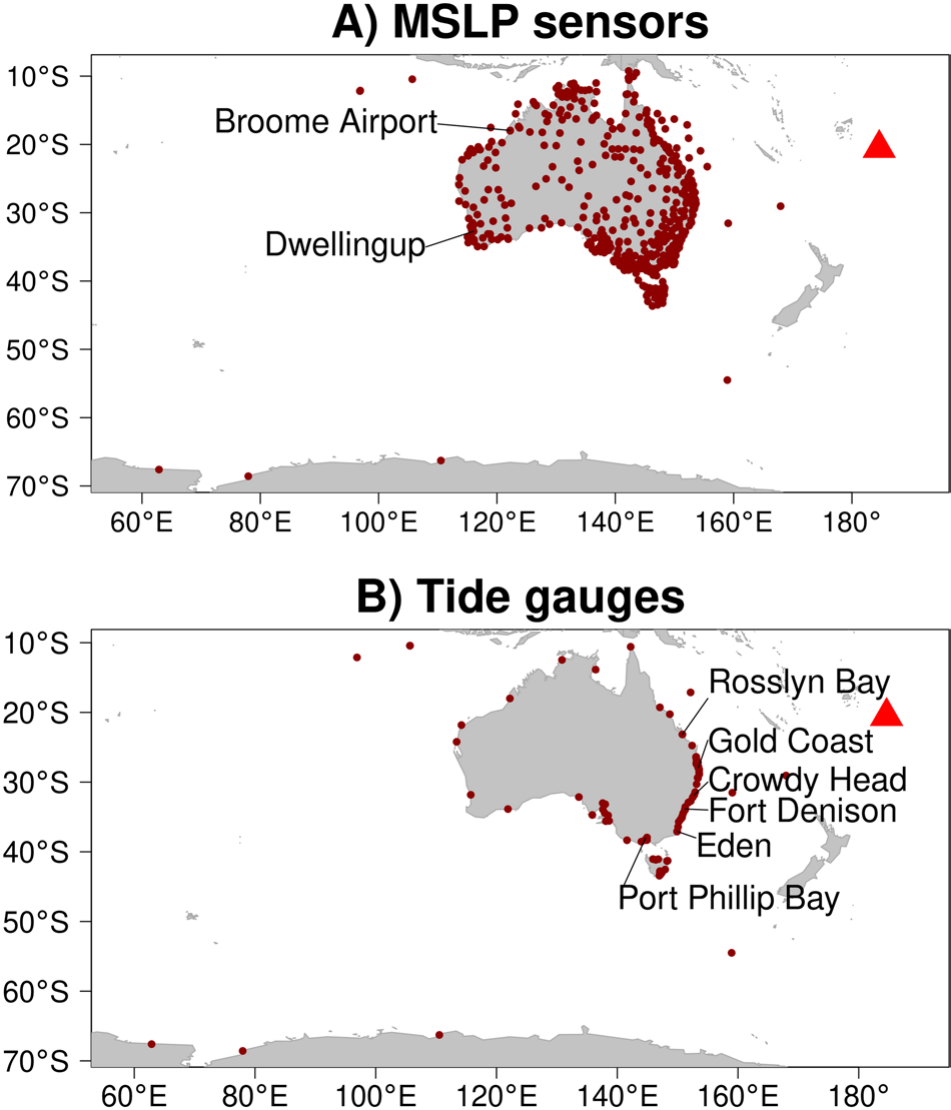
Locations of data (points) and the HTHH volcano (red triangle). (**A**) MSLP sensors. (**B**) tide gauges.

This study presents multi-site time series of mean sea level pressure (MSLP) and sea level around Australia during January 2022^8^. All the time series include data before and after the explosion, and the majority show evidence of the atmospheric wave (MSLP) or tsunami (sea level). After discarding some stations due to problematic data, as discussed below, the dataset includes (Table 1):

- 503 MSLP sensors from throughout Australia and its offshore territories (Fig. 1a).
- 103 tide gauges which have Australia-wide coverage (Fig. 1b) but with higher concentration in south-eastern Australia where the tsunami was larger overall.

**Table 1:** Summary of MSLP and sea level datasets included in this study.

Type	# Stations	Dataset ID	Provided by	Notes
MSLP	467	MSLPBOM	Bureau of Meteorology	Nationwide network, http://www.bom.gov.au/climate/data/stations/
MSLP	36	MSLPDES	Queensland (QLD) Department of Environment and Science	Network in QLD, https://www.qld.gov.au/environment/coasts-waterways/beach/storm/storm-sites
Sea level	22	DES	QLD Department of Environment and Science	Network in QLD, https://www.qld.gov.au/environment/coasts-waterways/beach/tide-sites
Sea level	19	MHL	Manly Hydraulics Laboratory	Network in New South Wales (NSW) including Lord Howe Island, https://www.mhl.nsw.gov.au/
Sea level	9	TAS	University of Tasmania	Short term tide gauge deployments for the Tasmania Tide Monitoring project^16^
Sea level	17	BOMPorts	Multiple Port Authorities (via Bureau of Meteorology)	Network in south-eastern Australia. Collected by a range of Port Authorities (Port Authority of NSW, TasPorts, Victorian Ports Corporation and Flinders Ports), http://www.bom.gov.au/oceanography/projects/ntc/ntc.shtml
Sea level	8	PANSW	Port Authority of NSW	Commercial data purchased by Geoscience Australia. Licence prohibits redistribution of raw tide gauge observations (not included herein) but permits redistribution of high-pass filtered time series. http://wavewindtide.portauthoritynsw.com.au/
Sea level	4	AAD	Australian Antarctic Division	Tide gauges at Macquarie Island, Mawson, Casey and Davis. https://www.antarctica.gov.au/about-us/contact/
Sea level	24	IOC	IOC sea level monitoring website	All stations provided to the Intergovernmental Oceanographic Commission of UNESCO (IOC) by the Bureau of Meteorology, http://www.ioc-sealevelmonitoring.org/

The “# Stations” column counts stations in the post-processed dataset (details in Methods). The “Dataset ID” appears in the post-processed metadata tables and is used to refer to subsets of stations from different data providers.

Compared with MSLP and sea level data for the HTHH volcanic tsunami that is already openly available^6,9,10^, the data herein^8^ greatly increases the density of observations near Australia.

The MSLP data was recorded at a network of barometers by the Bureau of Meteorology and the QLD Department of Environment and Science (Fig. 1a, Table 1). All these time series record MSLP at 1-minute intervals with occasional larger gaps due to missing data. All but two of the MSLPBOM time series span 8 days, while the MSLPDES time series all span 31 days.

The sea level data (Fig. 1b) was collected by a range of organisations (Table 1). Most tide gauges record sea level at 1-minute intervals (80 of 103) while the remainder use intervals between 2 and 10-minutes, with occasional larger gaps due to missing data. Most gauges include 20 or more days of data (73 of 103). They use a variety of vertical datums, often approximating either Australian Height Datum or a local lowest astronomical tide. No attempt was made to convert all the sea level data to a single vertical datum because the required information is not always available (although it is included in the original data for a high fraction of tide gauges). In some instances, the combination of data from multiple sources leads to two gauges being almost co-located, and in one case the same gauge is included twice but with different processing (smoothing and down-sampling) having been implemented by the data providers. In these cases, both datasets are included because they can provide insight into measurement uncertainties caused by instrumentation and processing (discussed below).

**Fig. 2:**
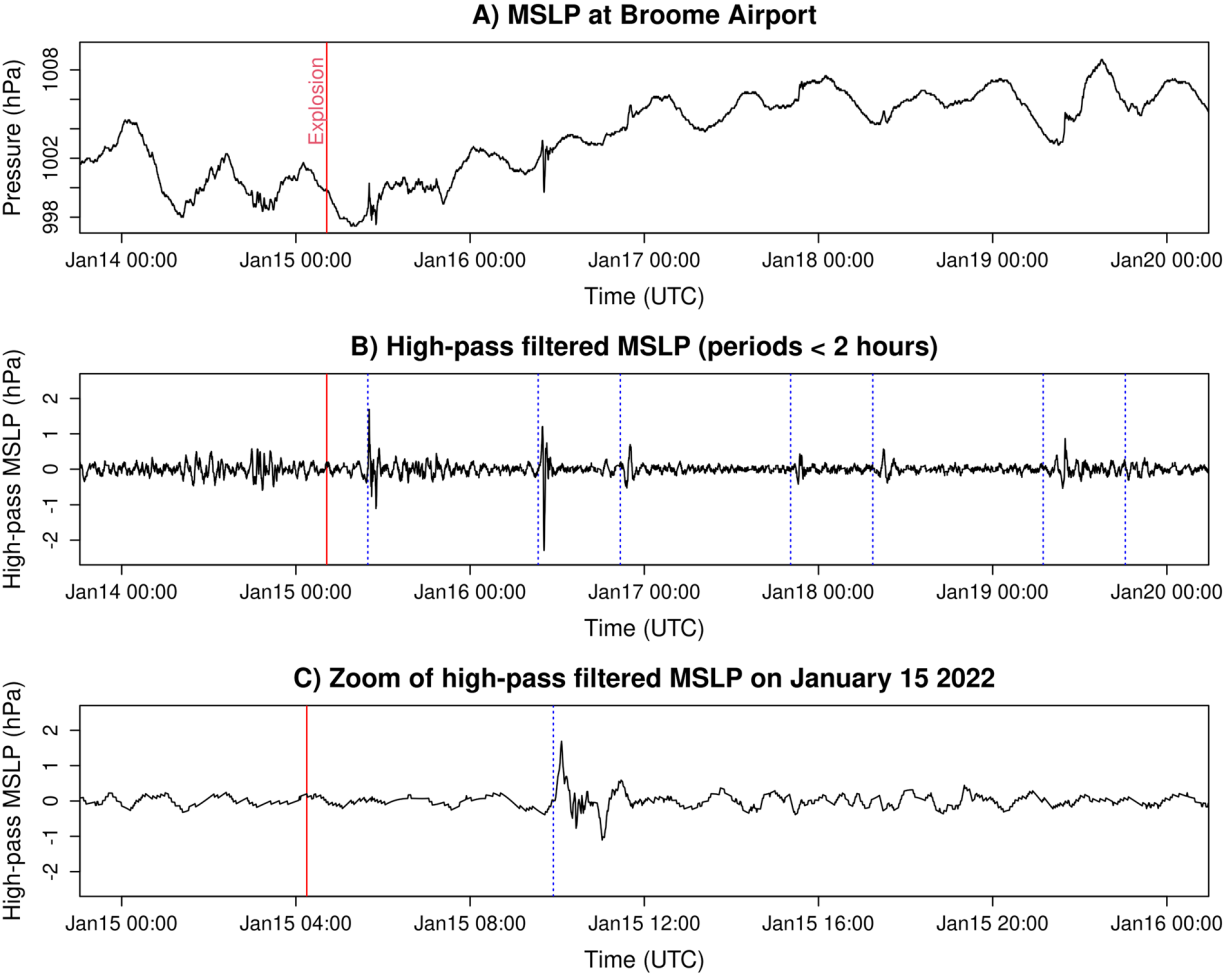
Example of MSLP data processing for Broome Airport (location in Fig. 1). (**A**) Original data from January 14–19 inclusive. (**B**) High-pass filtered MSLP, with spikes showing the effects of the volcano generated atmospheric Lamb wave. Dashed vertical lines show the theoretical arrival times for a Lamb wave travelling from the HTHH Volcano at constant speed (320 m/s) along a great circle path. (**C**) Zoom of the middle panel showing only January 15, with the Lamb wave arrival well matching the theoretical arrival time.

**Fig. 3:**
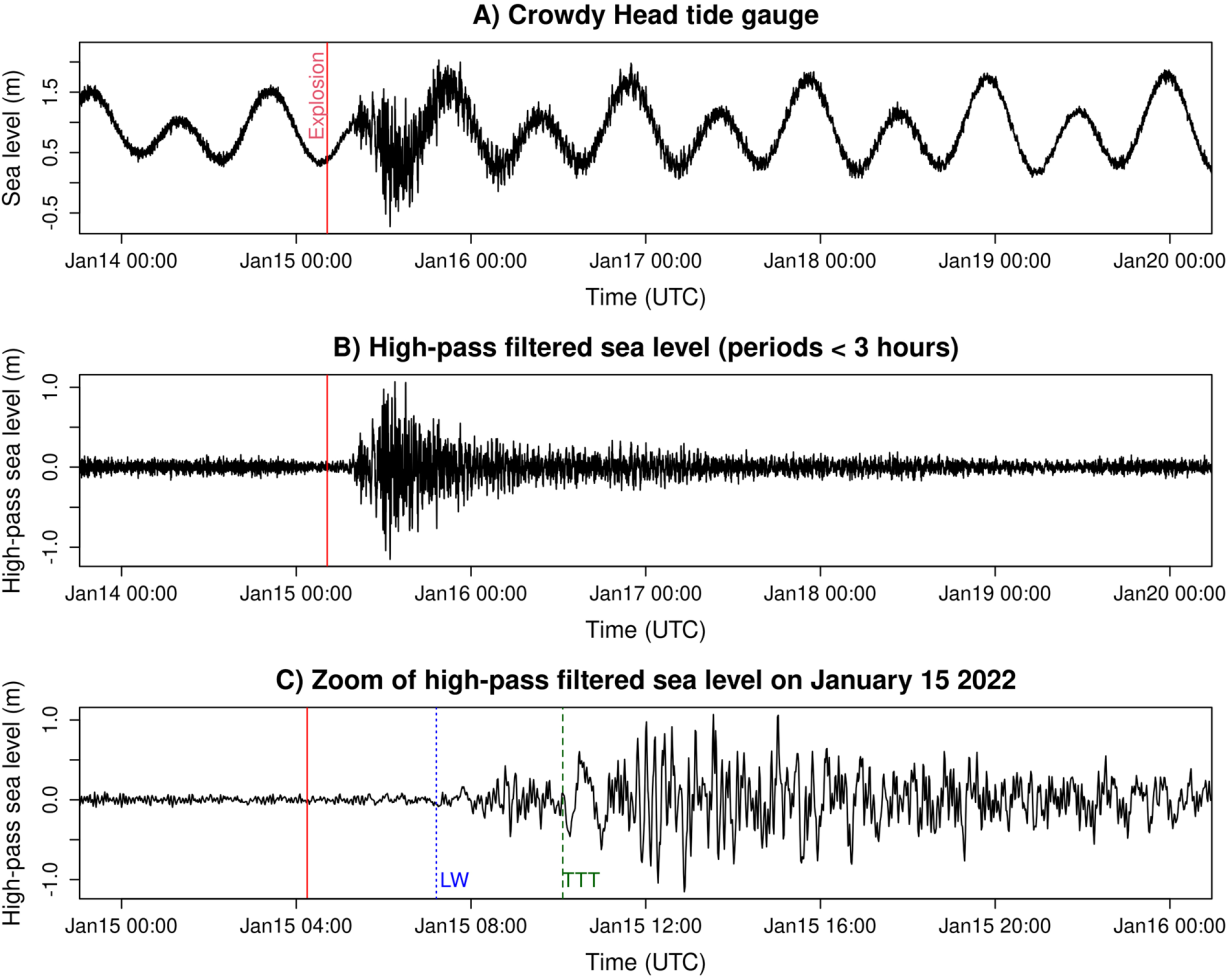
Example of sea level data processing for Crowdy Head (location in Fig. 1). (**A**) Original data from January 14–19 inclusive. (**B**) High-pass filtered sea level, with a clear tsunami signal following the main explosion. (**C**) Zoom of panel B showing only January 15, including the largest tsunami waves. The vertical line ‘LW’ shows the theoretical Lamb wave arrival time, while ‘TTT’ shows the tsunami travel time derived theoretically^11^ assuming oceanic propagation from the HTHH volcano.

The original MSLP and tide gauge time series are non-trivial to analyse because they employ multiple file formats and time zones, occasionally contain errors, and do not separate the effects of regular MSLP or sea level variations from the volcanic pressure wave or tsunami. Therefore, in addition to the original data, we provide post-processed datasets with a unified file format, consistent UTC time zone, and additional quality control. The post-processed data includes both raw time series and a high-pass filtered variant which better represents the atmospheric pressure wave (Fig. 2b,c) or tsunami (Fig. 3b,c). For a minority of tide gauges (8/103) licence constraints prevent redistribution of the original data, and only the high-pass filtered timeseries are provided (Table 1).

The high-pass filtered time series include wave periods less than 2 hours (for MSLP) or 3 hours (for sea level). These short period waves are often dominated by the atmospheric Lamb wave (Fig. 2b,c) or tsunami (Fig. 3b,c) and studies typically use similar high-pass filters to study their effects^1,7,10^. The short period waves are also generated by other processes, so are not zero amplitude prior to the HTHH volcanic explosion, but their amplitude varies greatly from site to site. At many sites the tsunami arrives earlier than would be expected for a long wave travelling from the HTHH volcano through the ocean (Fig. 3c), reflecting tsunami generation by the atmospheric pressure waves^6,7,11^.

The dataset^8^ includes post-processed time series, original time series (except where licence constraints prohibit redistribution), code used for post-processing, and figures used for quality control. In practice quality control was implemented by iteratively post-processing and creating figures to identify problematic data, which was then fixed by modifying the post-processing code or obtaining updated original data (details below). Because the archived files^8^ represent the final iteration, the post-processed time series should not evidence problems with the data. The aim is to provide an easy-to-use, transparent, and relatively comprehensive record of this rare volcanic pressure wave and tsunami in Australia.

## Methods

MSLP and sea level metadata, and associated time series, were extracted from the original data provided for the study (Table 1). All time zones were converted to UTC. The original metadata files sometimes included stations without corresponding time series, which were skipped in post-processing. Stations were also skipped if the graphical checks (discussed below) showed that, in the days following the volcanic explosion, data was entirely missing or strongly obscured by errors. Station metadata tables were written separately for the MSLP and tide gauge data and imported into Geographic Information Systems (GIS) to visually check the station locations.

### MSLP data: Processing and high-pass filter

All MSLP time series were plotted for quality control purposes, along with a high-pass filtered variant including wave periods less than 2 hours (Fig. 2). The 2-hour threshold is consistent with the long-period limit used in other studies that focus on the pressure wave^1,10^. Visual inspection showed seven stations had data gaps that strongly obscured the post-explosion atmospheric pressure wave. These stations were skipped in post-processing. Finally, the original MSLP and high-pass filtered time series were written to post-processed files.

The high-pass filter algorithm is as follows. Firstly, the data was limited to a 30-day period containing observations before and after the eruption (January 02–31 inclusive). This was transformed to a periodic series by subtracting a linear trend defined by the first and last data points, and then linearly interpolated to a uniform 15 second spacing. A discrete Fourier transform (DFT) was then applied, and spectra with periods shorter than the threshold were zeroed before applying an inverse DFT and adding back the linear trend. This defines the long-period component of the interpolated data. The latter was re-interpolated to the original data times, within the considered 30-day period, and finally subtracted from the original data to define the high-pass filtered time series. Examples are shown in Fig. 2b,c.

### Sea level data: Processing and high-pass filter

All tide gauge time series were plotted for quality control purposes, along with a high-pass filtered time series containing wave periods less than 3 hours (Fig. 3). The high-pass filter was identical to that used for the MSLP data but with a 3-hour threshold to match a previous study of tsunamis in Australia^12^. Visual inspection highlighted errors in seven time series, including localised spikes, spurious constant data, unrealistic high-frequency noise, or too many data gaps to usefully record the sea level. One record (Port Giles) was heavily contaminated by noise and completely removed. The others are partially edited within the post-processing scripts to remove spurious data. No interpolation was performed.

Two different approaches were tested to extract short-period waves from the tide gauge data, which better represent the tsunami. The first approach was the simple 3-hour high-pass filter described above. The second approach was similar except that astronomical tide predictions from the tide model TPXO9v5a^13^ were subtracted from the observed sea levels, before high-pass filtering to remove other long-period sea level variations. Graphical comparisons (available in the data repository) show negligible difference between these two approaches at all gauges. This is because the tidal predictions overwhelmingly contain wave periods longer than 3 hours, which are automatically removed by the high-pass filter. Thus, the simple 3-hour high-pass filter was written to the post-processed data files. This also ensures that users of post-processed data are not restricted by the tidal model’s non-commercial licence.

Sea level time series are included irrespective of whether they show a clear tsunami signal, because even the absence of a signal may give some insight into the tsunami dynamics. When the data licence does not permit release of raw tidal measurements, the post-processed sea level time series contain missing data, but the high-pass filtered time series are still included (Table 1).

## Data Records

The data record^8^ contains three top-level subfolders. Most of these include README.md files developed by the authors to further document the contents.

The folder “original” contains the original MSLP and tide gauge data, and metadata, obtained for this study from the data providers (Table 1). The data formats and time zones vary. Some of the time series include artefacts (e.g. excessive noise) as discussed above. To make the processing transparent we did not alter the time series in “original”, but instead, artefacts are treated in our scripts when producing the post-processed data. The tide gauge sub-folder includes a file README.md with some notes on the tide gauge configuration (where available). This often includes information on smoothing of sea levels by the tide gauges, which can affect the tsunami measurements (discussed in the Technical Validation section).

The folder “post-processed” contains post-processed data and plots derived from the data in “original”. The data has a consistent format and UTC time zone. The folder also includes all plots used for quality-control purposes. The key elements are:

- A metadata table describing the tide gauges is in “01_tide_gauge_locations.csv”.
- A metadata table describing the MSLP sensors is in “02_mslp_sensor_locations.csv”.
- Post-processed sea level time series are in the subfolder “01_tide_gauges”.
- Post-processed MSLP time series are in the sub-folder “02_mslp_sensors”.
- Figures are in the sub-folder “03_graphical_checks”.
- The names of stations that were included in the original metadata but not processed further (due to missing data or other quality control issues) are listed in files ‘ignored_mslp_sensors.txt’ and ‘ignored_tide_gauges.txt’

The folder “scripts_to_postprocess” includes all scripts used to post-process the data in “original” and produce outputs in “post-processed”. They are mostly written in R^14^.

- They are provided for transparency but do not need to be run to use the data.
- Because some tide gauge data was removed from the “original” folder for the final archive (due to licence restrictions), the scripts should not be re-run as-is.
  - Doing so will overwrite files in “post-processed” and remove gauges that could not be included in the archived “original” data folder.
  - To prevent this, change the variable OUTPUT_DIR in the script global_variables.R before running the code.

## Technical Validation

### Validation of the MSLP data

The original MSLP data from the Bureau of Meteorology includes a quality flag for each observation. For the post-processed observations, the vast majority (99.3%) are classified as ‘Quality Controlled and Acceptable’. The remaining observations are classified as either ‘Quality Controlled and Suspect’ (0.066%) or ‘Inconsistent with other known information’ (0.014%) but were not identified as problematic in the tests below.

Plots of the MSLP data and its high-pass filtered variant (Fig. 2) were made for every station to help detect problematic data. For two stations all MSLP data was missing. Others had no data available during the period of the Lamb wave, or data gaps that substantially obscured the Lamb wave, and one had a large vertical discontinuity several days after the volcano. These were skipped by our post-processing scripts (noted in the “ignored_mslp_sensors.txt” file) but are included in the original data.

The high-pass filtered MSLP time series were also checked for consistency with expectations for the Lamb wave, considering both arrival times and amplitudes. To first approximation the initial Lamb wave should behave as a wave-front expanding radially from the HTHH volcano near the speed of sound, with form similar to Fig. 2c, and amplitude that decreases as the length of the wave-front increases^1,7,15^. By checking if the high-pass filtered time series match these expectations we can potentially detect outliers associated with errors in the station location, time zone, or the MSLP data itself. Our calculations assume a Lamb wave speed of 320 m/s along great circle paths, and an explosion time of 04:15 UTC, which is a simplification of the globally observed Lamb wave behaviour but adequate for technical validation^7^. The maximum high-pass filtered MSLP value was selected from a time window ± 2 hours of the theoretical arrival time. Although the Lamb wave should occur within a smaller time-window, the larger window is preferable for technical validation as it could enable spurious time-offsets in the data to be detected.

**Fig. 4:**
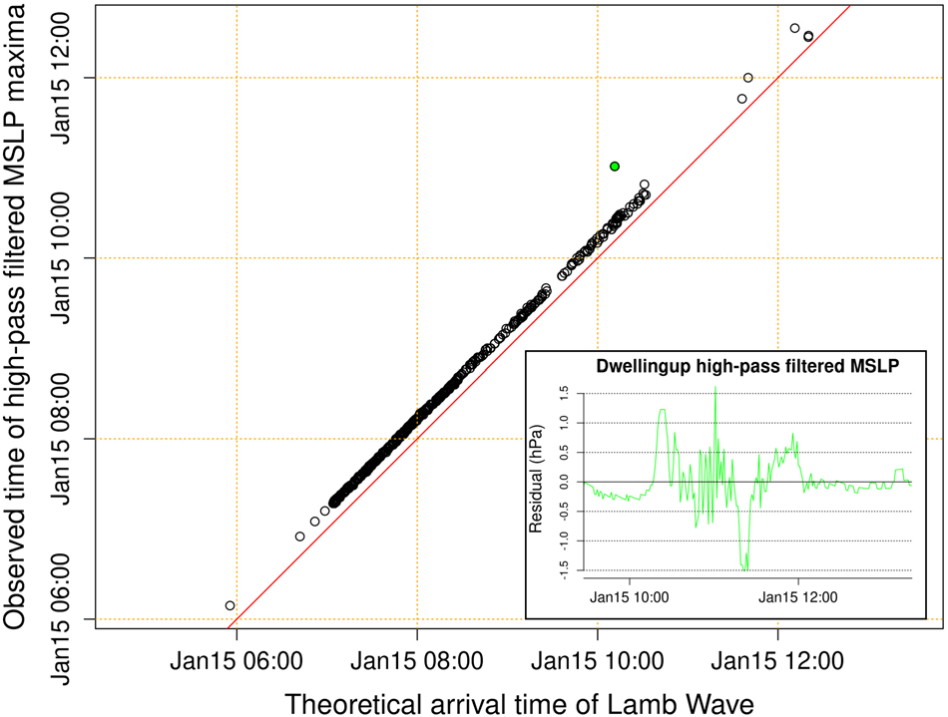
Comparison of the theoretical Lamb wave arrival time and the observed time of the high-pass filtered MSLP maxima. The red line is y = x. The observed maxima occur shortly after the theoretical arrival time, consistent with expectations for the Lamb wave. The outlier (green) is at Dwellingup with location in Fig. 1.

The high-pass filtered MSLP maxima occurs at the expected time, soon after the theoretical Lamb wave arrival time (Fig. 4). For all but one station the time difference is less than 25 minutes. Visual inspection showed the single outlier (Dwellingup, time difference of 45 min) was due to unusually strong MSLP oscillations, observed after the leading Lamb wave peak, which our algorithm identified as the maxima (Fig. 4). Similar oscillations are observed at other stations with smaller magnitude (Fig. 2c) and we do not have evidence that this measurement is incorrect.

**Fig. 5:**
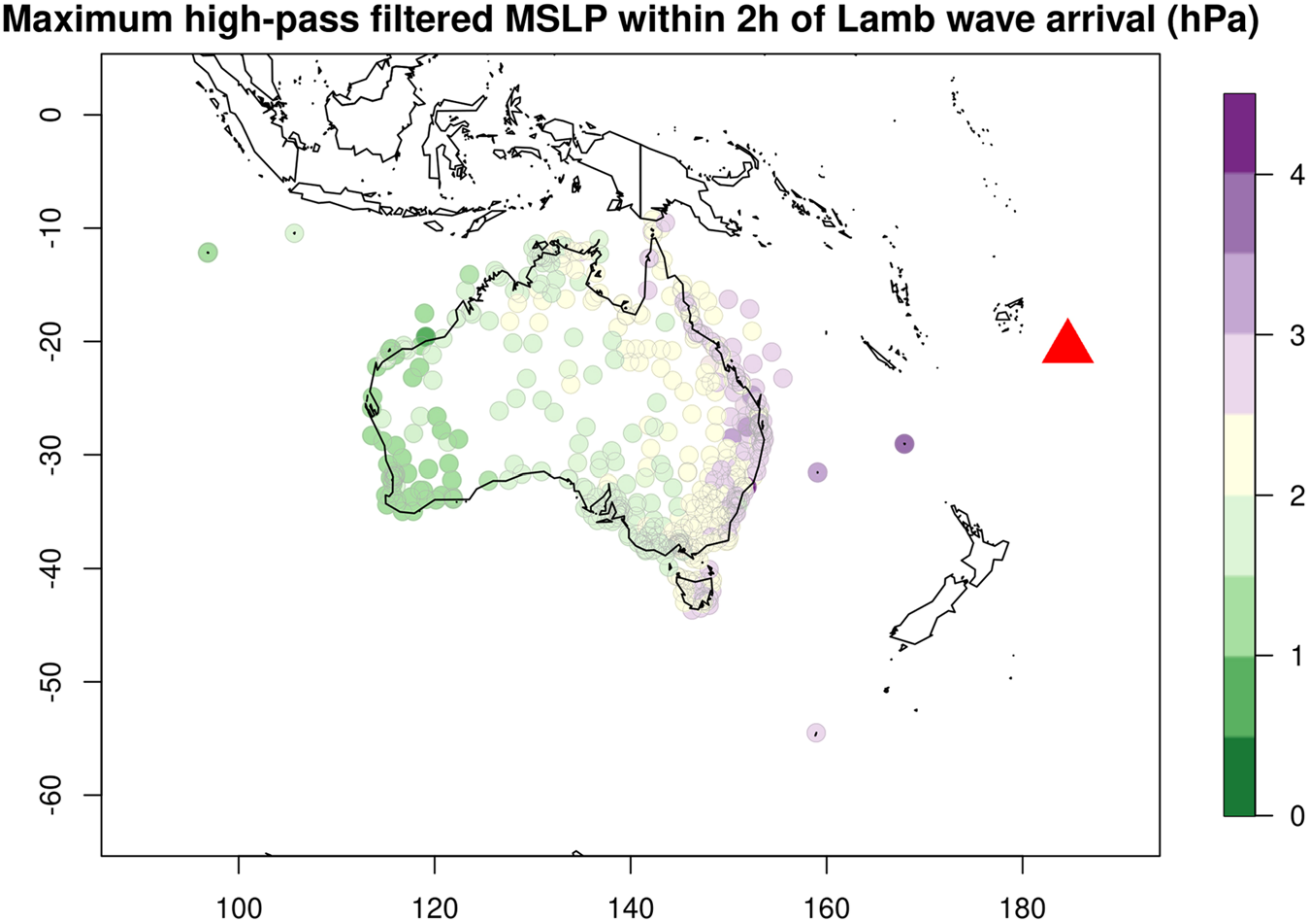
Maximum of the high-pass filtered MSLP within ±2 h of the theoretical Lamb wave arrival time. It shows the expected tendency to decrease with distance from the HTHH volcano (red triangle).

The high-pass filtered MSLP maxima also show a general decrease with distance from the HTHH volcano, without obvious outliers (Fig. 5). This is expected near Australia where the circular initial Lamb wave front is lengthening with distance travelled, which causes spreading of the wave energy^1,15^.

### Validation of the sea level data

Graphical checks of the sea level time series and high-pass filtered variants were undertaken for January 14 – January 18 inclusive. This highlighted major artefacts in some time series that were addressed as described in the Methods. Graphical checks of the station locations also led to some large errors in tide gauge locations being fixed: one due to an incorrect sign of latitude; one where a site location had been substituted for another. These corrections were reported to the data providers and then corrected in the original data (so there are no inconsistencies in the provided data archive). Errors of the order of 10 m likely remain in some station locations, due to coordinates being provided with limited precision, but we did not have independent information to correct these.

Measured sea levels were compared with predictions from the astronomical tide model TPXO9.v5a^13^. This provides an opportunity to detect errors such as an incorrect time zone conversion or a large error in the gauge coordinate, although some differences are expected due to meteorological effects and hydrodynamic processes unresolved in the tidal model. At most sites the observations agree well with the TPXO9.v5a tidal predictions (all figures available in the data archive^8^). At sites showing large differences we checked the deviation seemed reasonable given the site location, assuming the tidal model might not resolve sites in estuaries or near extensive shallow bathymetry. For example, the tidal model overestimates the tide range in Port Phillip Bay (Fig. 1) which is substantially attenuated compared to the nearby open coast and includes gauges at Fawkner Beacon, Hovell Pile, and Queenscliffe.

**Fig. 6:**
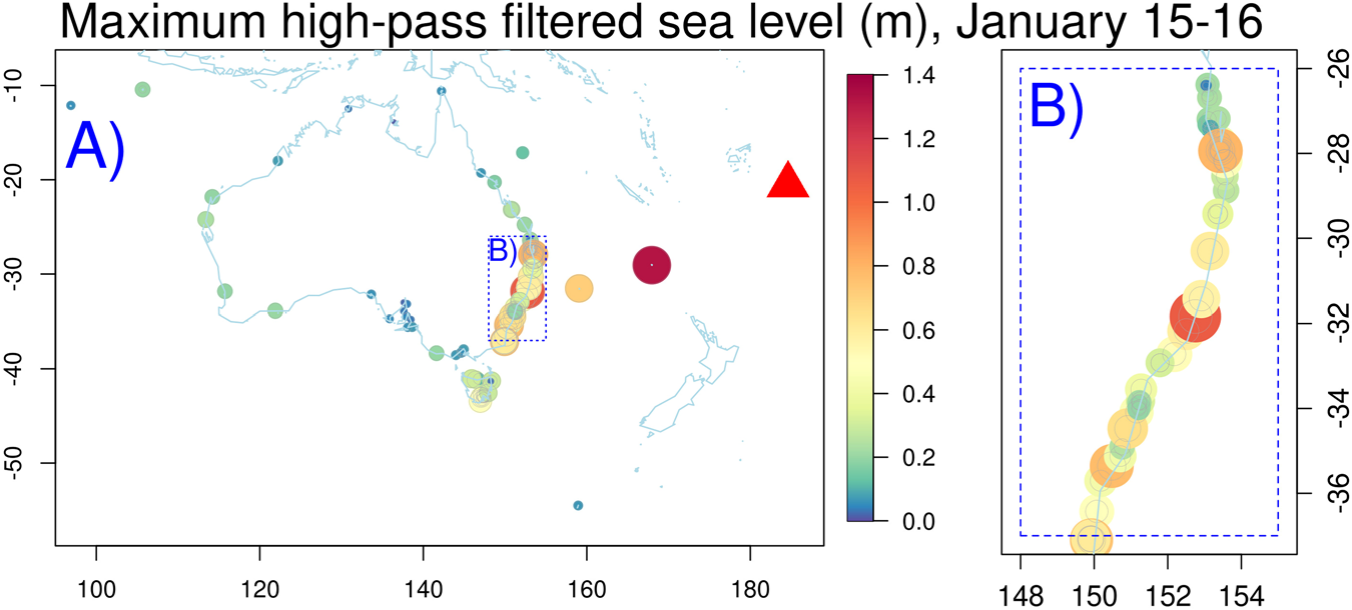
Maximum of the high-pass filtered sea level on January 15-16. (**A**) Large scale with red triangle showing the HTHH volcano location. (**B**) Zoom in eastern Australia. The tendency for higher values around south-eastern Australia is consistent with expectations for the tsunami.

The high-pass filtered sea level time series are expected to show evidence of the tsunami on January 15–16 at many sites, particularly closer to the HTHH volcano. Figure 6 shows the maximum high-pass filtered observations are broadly consistent with expectations, without obvious outliers. The tsunami is largest overall in south-eastern Australia and nearby offshore islands. The relatively dense observations in south-eastern Australia show significant tsunami size variability, consistent with observations of other tsunamis on this coast^12^, reflecting complex interactions of the tsunami with the variable coastal morphology.

Further technical validation was undertaken by comparing measurements at pairs of nearby tide gauges. Nearby gauges should exhibit similar sea levels in the absence of measurement biases. However, in practice there are biases due to the tide gauge configuration and/or post-processing by data providers. Our dataset includes one location (at Fort Denison in Sydney) where two time series were derived from a single gauge; a PANSW time series that records the tsunami at 1-min intervals (each an average of 12 measurements of 5-second average sea level), and a 6-min BOMPorts time series that was derived from the latter by smoothing as routinely implemented by the data providers. The dataset also includes five locations where two neighbouring instruments (<350 m spacing) record the sea level at 1-minute intervals but measure the sea level with different degrees of smoothing, either by design or choice of configuration options. Although details of the tide gauge configuration or post-processing are not available for every site, the potential for bias can be illustrated by comparing nearby gauges.

**Fig. 7:**
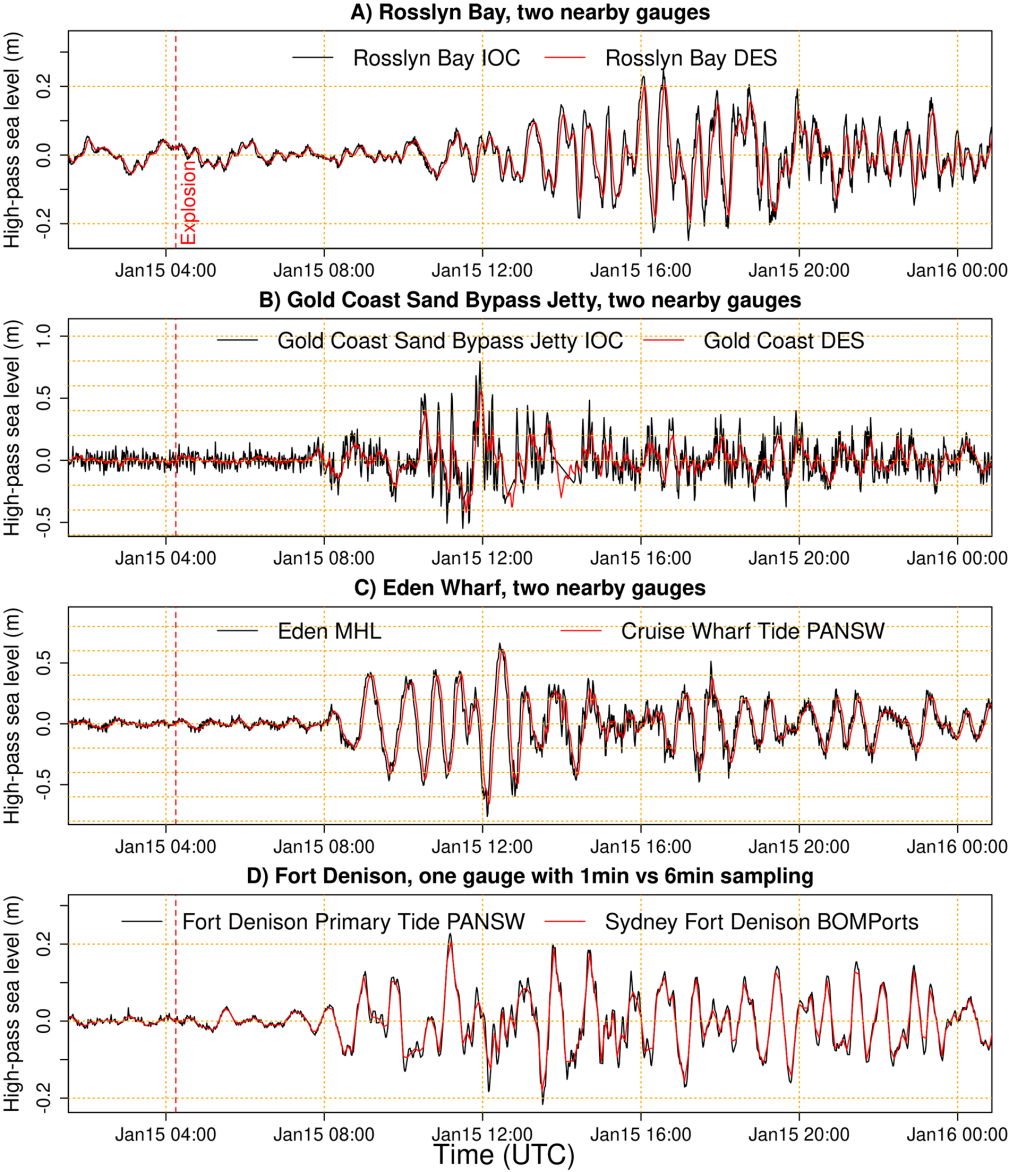
High-pass filtered sea level observations for pairs of nearby tide gauges following the HTHH volcano explosion, with locations in Fig. 1. (**A**) Gauges separated by 2 m at Rosslyn Bay; (**B**) Gauges separated by 2 m at the Gold Coast Sand Bypass Jetty; (**C**) Gauges separated by 320 m near Eden Cruise Wharf; (**D**) One gauge at Fort Denison where the 6-min BOMPorts record is derived from the 1-min PANSW record by smoothing.

Figure 7 compares high-pass filtered sea levels at four pairs of nearby gauges (<350 m spacing) during the initial stages of the HTHH volcano tsunami. Panels A-C represent different yet nearby gauges, whereas panel D represents 6-min and 1-min data derived from a single gauge. We deliberately chose pairs of gauges with relatively large differences and a clear tsunami signal, to better illustrate the potential for artefacts in tsunami measurements.

To first order all pairs of nearby gauges have similar high-pass filtered time series (Fig. 7). But significant differences occur at the Gold Coast site which is located on a high-energy beach and exhibits prominent short period waves (Fig. 7b). Here both gauges report data at 1-minute intervals, but the smoother gauge internally averages the measurements over a 4-minute interval before storing, leading to apparent differences in the tsunami maxima of 56 vs 80 cm. That difference is relatively extreme and not representative of most sites in our dataset, which are less influenced by short period waves. However, at all sites there are differences between gauges, with the smoother gauge showing attenuation of shorter periods and some reduction in the tsunami maxima and minima (Fig. 7). Similar distortions of the tsunami signal are expected to be common, depending on the tide gauge configuration, and should be considered when using sea level measurements to study the tsunami.

## Data Availability

All code is provided within the data archive^8^, and separately at https://github.com/GeoscienceAustralia/ptha/tree/master/misc/hunga_tonga_data_paper.
